# Autologous chondrocyte grafting promotes bone formation in the posterolateral spine

**DOI:** 10.1002/jsp2.1001

**Published:** 2018-03-23

**Authors:** J. Alex Sielatycki, Masanori Saito, Masato Yuasa, Stephanie N. Moore‐Lotridge, Sasidhar Uppuganti, Juan M. Colazo, Alexander A. Hysong, J. Patton Robinette, Atsushi Okawa, Toshitaka Yoshii, Herbert S. Schwartz, Jeffry S. Nyman, Jonathan G. Schoenecker

**Affiliations:** ^1^ Department of Orthopaedics and Rehabilitation Vanderbilt University Medical Center Nashville Tennessee; ^2^ Department of Orthopaedic Surgery Tokyo Medical and Dental University Tokyo Japan; ^3^ Department of Pharmacology Vanderbilt University Nashville Tennessee; ^4^ Vanderbilt University School of Medicine Nashville Tennessee; ^5^ Department of Biomedical Engineering Vanderbilt University Nashville Tennessee; ^6^ Center for Bone Biology Vanderbilt University Medical Center Nashville Tennessee; ^7^ Department of Veterans Affairs Tennessee Valley Health Care System Nashville Tennessee; ^8^ Department of Pathology, Microbiology, and Immunology Vanderbilt University Medical Center Nashville Tennessee; ^9^ Department of Pediatrics Vanderbilt University Medical Center Nashville Tennessee

**Keywords:** bone formation, bone‐morphogenic protein, fracture callus, hypertrophic chondrocytes, iliac crest bone graft, ossification, posterolateral spinal fusion, vascular endothelia growth factor

## Abstract

**Background context:**

Pseudarthrosis following spinal fusion remains problematic despite modern surgical and grafting techniques. In surgical spinal fusion, new bone forms via intramembranous and endochondral ossification, with endochondral ossification occurring in the hypoxic zones of the fusion bed. During bone development and fracture healing, the key cellular mediator of endochondral ossification is the hypertrophic chondrocyte given its ability to function in hypoxia and induce neovascularization and ossification. We therefore hypothesize that hypertrophic chondrocytes may be an effective bone graft alternative.

**Purpose:**

Spinal fusion procedures have increased substantially; yet 5% to 35% of all spinal fusions may result in pseudoarthrosis. Pseudoarthrosis may occur because of implant failure, infection, or biological failure, among other reasons. Advances in surgical techniques and bone grafting have improved fusion; however pseudarthrosis rates remain unacceptably high. Thus, the goal of this study is to investigate hypertrophic chondrocytes as a potential biological graft alternative.

**Methods:**

Using a validated murine fracture model, hypertrophic chondrocytes were harvested from fracture calluses and transplanted into the posterolateral spines of identical mice. New bone formation was assessed by X‐ray, microcomputed tomography (μCT), and *in vivo* fluorescent imaging. Results were compared against a standard iliac crest bone graft and a sham surgery control group. Funding for this work was provided by the Department of Orthopaedics and Rehabilitation, the OREF (Grant #16‐150), and The Caitlin Lovejoy Fund.

**Results:**

Radiography, μCT, and *in vivo* fluorescent imaging demonstrated that hypertrophic chondrocytes promoted bone formation at rates equivalent to iliac crest autograft. Additionally, μCT analysis demonstrated similar fusion rates in a subset of mice from the iliac crest and hypertrophic chondrocyte groups.

**Conclusions:**

This proof‐of‐concept study indicates that hypertrophic chondrocytes can promote bone formation comparable to iliac crest bone graft. These findings provide the foundation for future studies to investigate the potential therapeutic use of hypertrophic chondrocytes in spinal fusion.

## 1 INTRODUCTION

Spinal fusion procedures have increased significantly over the past 10 years.^1^, ^2^ While a successful spinal fusion rates have improved with modern implant and grafting techniques, complications can arise, with approximately 5% to 35% of all spinal fusions developing pseudoarthrosis.^2^, ^3^, ^4^, ^5^, ^6^, ^7^ Pseudoarthrosis, or nonunion, may lead to increased patient morbidity such as continued pain, segmental instability, and need for revision surgery. Additionally, revision surgeries following the development of a pseudoarthrosis are costly to the health care system, averaging $41 631 per revision.^8^ Pseudoarthrosis can develop for multiple reasons including, but not limited to, biomechanical failure of an implant, inadequate fusion bed preparation, infection of the fusion site, or biological failure of the bone. Thus, advances in surgical techniques and graft advancements are being actively investigated in order to reduce the rates of pseudoarthrosis.

Currently, autogenous bone graft (autograft) harvested from the iliac crest, commonly known as an iliac crest bone graft (ICBG), remains the gold standard for augmenting spinal fusion.^9^ Although ICBG demonstrates high fusion rates, its use is hampered by donor site morbidity and limited supply—particularly for multilevel fusions.^9^, ^10^, ^11^ Thus, there is a need for a bone graft alternative that can be clinically available in large quantities, provide ample bone formation, and subsequently result in low pseudoarthrosis rates and limited adverse side effects.

**Figure 1:**
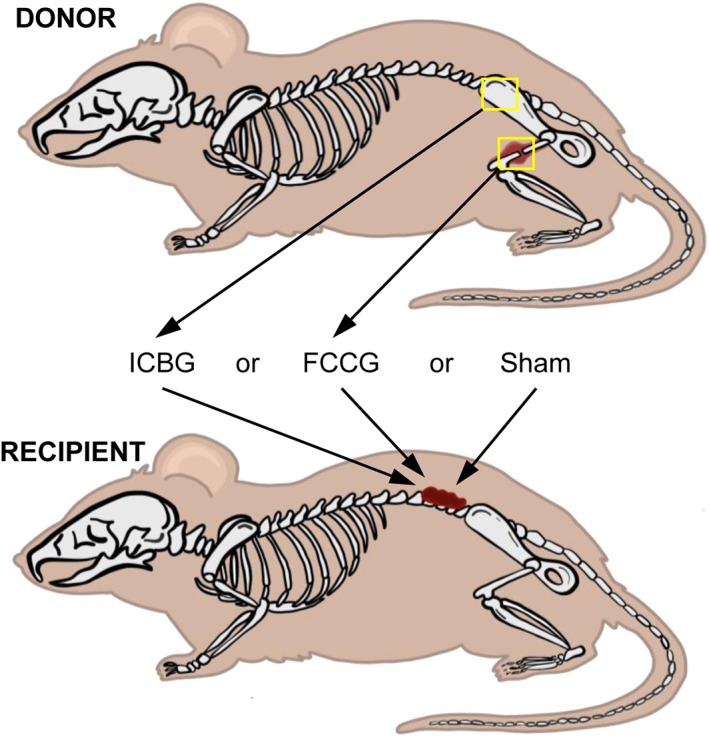
Experimental design—use of fracture callus chondrocyte graft (FCCG) as compared to iliac crest bone graft (ICBG) for promoting bone formation in the posterolateral spine. To test the hypothesis, ICBG and FCCG were harvested from a single donor mouse. Grafts were then individually transplanted into the posterolateral gutters of a genetically identical recipient mouse. A sham surgery with no graft implantation was used as a control. *N* = 10 per experimental group*Note*: Inspiration for image taken from BSIP/Universal Imaging Group/Getty Images

In spinal fusion, new bone formation has been previously described to occur via intramembranous and endochondral ossification—processes similar to long bone fracture healing.^10^, ^12^, ^13^, ^14^, ^15^, ^16^ The key cellular mediator of endochondral ossification is the hypertrophic chondrocyte.^17^ Along with their unique ability to survive in hypoxia, hypertrophic chondrocytes induce neovascularization and ossification through the release of vascular endothelial growth factor (VEGF), vesicles of hydroxyapatite, and bone‐morphogenic proteins (BMPs).^5^, ^17^, ^18^, ^19^ For these reasons, we hypothesize that hypertrophic chondrocytes offer a potential graft alternative for promoting spinal fusion. To test this hypothesis, hypertrophic chondrocytes were harvested from a soft fracture callus at day 10 postfracture and surgically implanted into the posterolateral spinal gutters of a genetically identical mouse recipient. Following implantation, the ability of these fracture callus chondrocyte grafts (FCCGs) to drive posterolateral bone formation was assessed and compared with either standard iliac crest bone graft (ICBG) or a sham implantation surgery control group (Figure 1).

## 2 MATERIALS AND METHODS

All animal procedures were reviewed and approved by the Institutional Animal Care and Use Committee (IACUC) of Vanderbilt University Medical Center (M1600140).

### 2.1 Graft harvesting (FCCG and ICBG)

**Figure 2:**
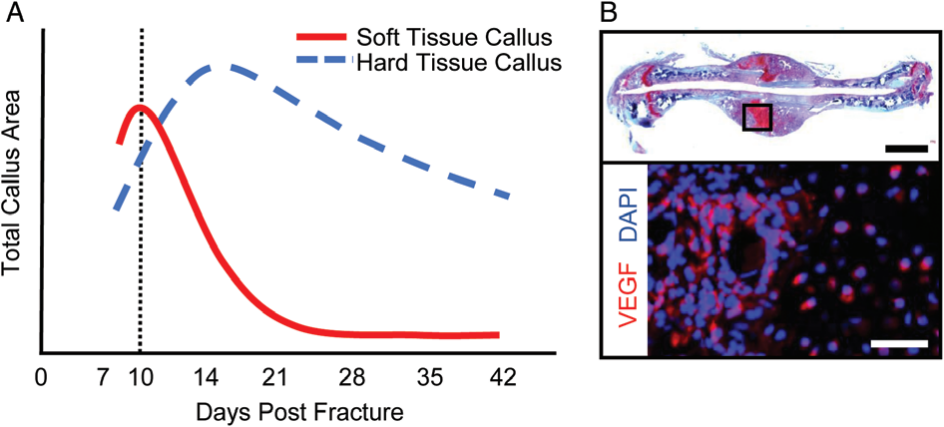
Optimal timing for fracture callus chondrocyte graft (FCCG) isolation. Previous longitudinal investigations of fracture callus size and composition^20^ have demonstrated (A) maximal soft tissue callus volume at 10 days postfracture (red line) and maximal hard tissue callus between 14 and 21 days postfracture (blue dashed line). (B) at 10 days postinjury, hypertrophic chondrocytes found within the soft tissue callus are producing VEGF‐(A) Top panel: Scale bar = 1 mm. Bottom panel: Scale bar = 200 μm

Male C57BL/6J mice were purchased from Jackson Laboratory and housed at Vanderbilt University in a 12‐h light/dark cycle with food and water provided *ad libitum*. At 8 weeks of age, an open femur fracture model, previously developed by our lab,^17^, ^20^ was performed. Following adequate anesthesia and analgesia, a 10 to 12 mm long medial incision was made to expose the mid‐shaft of the femur. The femur was then fractured in a controlled manner by scoring the bone with a beaver blade before inducing a clean break. The transverse fracture was stabilized with the intramedullary placement of a 30‐gauge needle, to induce a larger soft‐tissue fracture callus, as compared with needles of a larger size (23G) with more stiffness (unpublished results); thereby allowing for a more efficient harvest. The incision was then closed using 5‐0 nylon sutures. Mice received analgesics every 12 h for 3 days following the fracture procedure to minimize pain and discomfort. Ten days following the fracture, when the soft‐tissue callus was largest and amply expressing VEGF (Figure 2), the mice were sacrificed by CO_2_ inhalation. At this time, the FCCG was harvested, along with an ICBG, for the subsequent transplantation into the posterolateral spine of syngeneic mice. Harvested grafts were standardized by volume and a 2 × 2 × 2‐mm section was obtained for implantation.

### 2.2 Murine posterolateral spinal bone formation model

**Figure 3:**
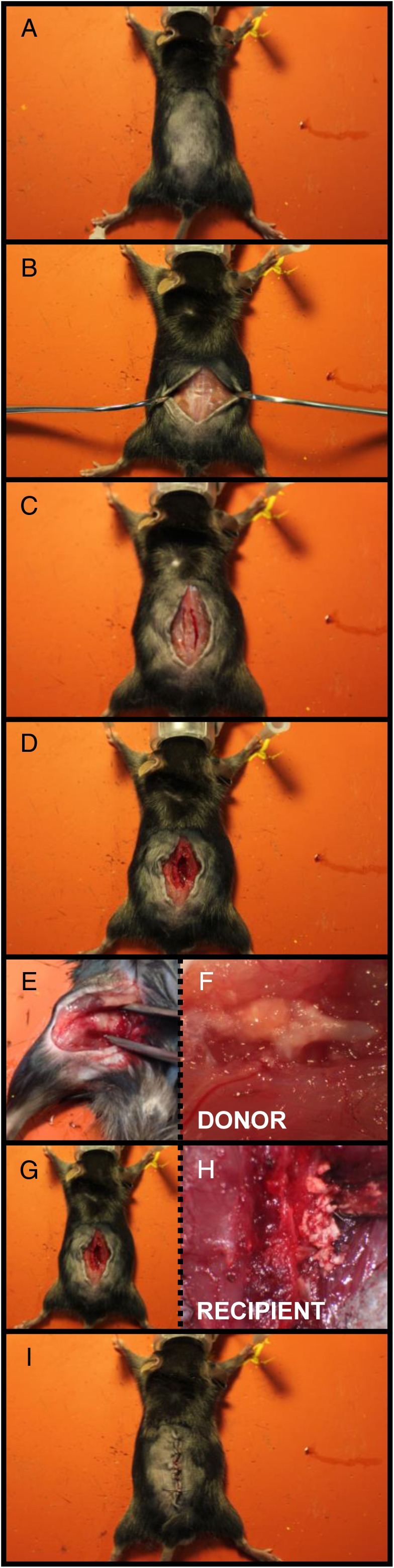
Murine posterolateral spinal fusion model. (A) Removal of dorsal hair to prepare incision site. (B) and (C) Midline incision followed by exposure of the dorsolumbar fascia and perispinal musculature. (D) Sub periosteal dissection to expose the transverse processes of the L3 to L5 vertebrae. (E) Isolation of fracture callus chondrocyte graft (FCCG) from donor mouse. (F) Magnified view of a soft‐tissue fracture callus for hypertrophic chondrocyte collection. (G) and (H) transplantation of graft (FCCG or iliac crest bone graft [ICBG]) into the posterolateral gutters of recipient mouse. (I) Incision closure

The purpose of this surgery model was to determine the capacity of FCCG or ICBG to promote bone formation following implantation to the posterolateral lumbar spine. Immediately following FCCG and ICBG harvesting, posterolateral lumbar surgeries were performed on separate, yet genetically identical, male C57BL/6J mice from Jackson Laboratory at 8 weeks of age (Figure 3). Following adequate anesthesia and analgesic, the dorsal fur was removed (Figure 3A) and a midline incision was made through the skin and dorsolumbar fascia to expose the perispinal musculature (Figure 3B,C). Subperiosteal dissection was carried out using a beaver blade to expose the transverse processes of the L3 to L5 vertebrae prior to decortication (Figure 3D). The laminae and spinous processes were then decorticated with a beaver blade. The previously harvested FCCG or ICBG was then transplanted into the posterolateral gutters (Figure 3E‐H). Following this, the internal fascia was closed with absorbable monofilament suture while the skin was closed with 5‐0 nylon suture in a simple interrupted fashion (Figure 3I). Mice were then transferred to their respective cages and monitored until they regained normal ambulation. Analgesic was administered every 12 h for 3 days following surgery to minimize pain and discomfort.

### 2.3 Assessment of posterolateral bone formation

#### 2.3.1 Radiographical assessment and quantification

To assess the development of bone formation between the transverse processes, digital radiographs were obtained longitudinally (4 s, 35 kV) beginning day one postsurgery and then weekly until 6 weeks postsurgery (Faxitron, Tucson, Arizona). Mice were placed in the prone position, aligning the spine vertically within the imaging plane. Images were used to quantify the amount of bone formation by 3 blinded independent observers. For each image, the area surrounding the transverse processes of vertebrae L3 through L5 was selected (Figure S1A, Supporting information). The 6 selected areas were then scored for the amount of calcification present, with a score of “0” representing ≤25% of the total area becoming calcified, a score of “1” representing 26% to 50% of the total area becoming calcified, a score of “2” representing 51% to 75% of the total area becoming calcified, and a score of “3” representing >75% of the total area becoming calcified. The sum of the 6 boxes was then recorded per observer per mouse (Figure S1B,C). Inter‐ and intraobserver error was assessed through the use of kappa statistics. On average, observers were found to be in fair to moderate agreement (*κ* = 0.255‐0.489) per the Landis and Koch criteria. Additionally, when rescored with more than 7 days between analyses, intraobserver variability was found to be moderate with observers being in slight to moderate agreement (*κ* = 0.087‐0.481). Further assessment of individual observer scores over time (ie, the slope of the line) were found to have no statistical difference between observers for any experimental group as measured by the analysis of the covariance (ANCOVA). FCCG—*F* = 0.072, df = 2, *P* = .930; ICBG—*F* = 0.021, df = 2, *P* = .979; Sham surgery—*F* = 0.283, df = 2, *P* = .754.

#### 2.3.2 *In vivo* fluorescent imaging of bone formation

To assess new bone formation, OsteoSense 800 (NEV11105, PerkinElmer, Shelton, Connecticut), an NIR‐labeled, targeted fluorescent bisphosphonate was used to visualize areas of new calcification. A total of 24 h before imaging, a representative mouse per group was injected intraperitoneally with 2 nmol of OsteoSense 800 in a total volume of 100 μL. A Pearl® small animal imaging system and image studio software (LI‐COR Biotechnology, Lincoln, Nebraska) were utilized to measure *in vivo* fluorescence at an excitation wavelength of 780 nm and an emission wavelength of 805 nm.

#### 2.3.3 Microcomputed tomography (μCT) analysis

To further assess bone formation qualitatively, mice were sacrificed 6 weeks postsurgery and 3‐dimensional (3D) renderings of the posterior lumbar region were generated using μCT (μCT 40, Scanco Medical AG, Bassersdorf, Switzerland). μCT images of the spine from the thoracic to the sacral region were acquired using a polychromatic X‐ray source with peak beam voltage at 55 kVp and tube current of 145 μA. The sample acquisition settings were as follows, 1024 samples per 500 projections per 180° rotation of the sample tube holder and each projection lasting 232 ms, that is, integration time. The raw image slices, with an isotropic voxel size of 20 μm, were reconstructed using Scanco OpenVMS software (v8.4). Postreconstruction, the volume of interest containing the posterior elements of L3 to L5 was contoured by transecting the pedicles within a 5.93‐mm diameter tube. The calcified tissue was segmented from the soft tissue using a relatively low global threshold of 150 per mile of the X‐ray attenuation coefficient without a Gaussian noise filter. The Scanco evaluation software v6.6 also provided component labeling (CL) function with rank 1 to 1 in order to remove any small noisy speckles that were not connected to the main structure. The CL ranked, segmented image file was used to represent a 3D rendering of the L3 to L5 spine using Scanco 3D viewer v4.0‐4.

#### 2.3.4 Histological analysis

To assess the fracture calluses isolated from the donor mouse a subset of femurs were isolated, decalcified, processed, and embedded in paraffin prior to sectioning at 10 days following fracture injury, when the soft tissue callus was largest. Histological sections through the fracture callus were stained for the presence of VEGF.

#### 2.3.5 Immunofluorescent staining of VEGF

Following deparaffinization, slides were hydrated and processed for antigen retrieval using a solution of 0.1 M citric acid and 0.1 M sodium citrate. Slides were then heated for 2 min, cooled to room temperature, and washed with Tris‐buffer saline before blocking with a solution of 5% BSA and 10% goat serum. Immunostaining was performed with antimouse VEGF‐A (1:200, Abcam 46 154, Cambridge, Massachusetts) antibody overnight at 4°C. Slides were then washed, incubated with 10 μg/mL AlexaFluor‐647 anti‐rabbit secondary antibody (Life Technologies 792514, Grand Island, New York) in blocking buffer for 1 h at room temperature, and counterstained with DAPI. All microscopic images were obtained on a Zeiss Axio Imager A.1 (ZEISS, Oberkochen, Germany).

### 2.4 Statistical analysis

Inter‐ and intraobserver variability, for quantification of calcification surrounding the transverse processes, was assessed using kappa statistics and interpreted with the Landis and Koch criteria.^21^ Variability in the score over time between observers was assessed through an ANCOVA. Analysis of bone formation between groups at each time point was assessed by a nonparametric 2‐way analysis of variance (ANOVA), corrected for multiple comparisons. Statistical analysis was conducted in GraphPrism V6 (La Jolla, California) or STATA V14.2 (College Station, Texas).

## 3 RESULTS

### 3.1 Implantation of posterolateral hypertrophic chondrocytes (FCCG) promotes bone formation

**Figure 4:**
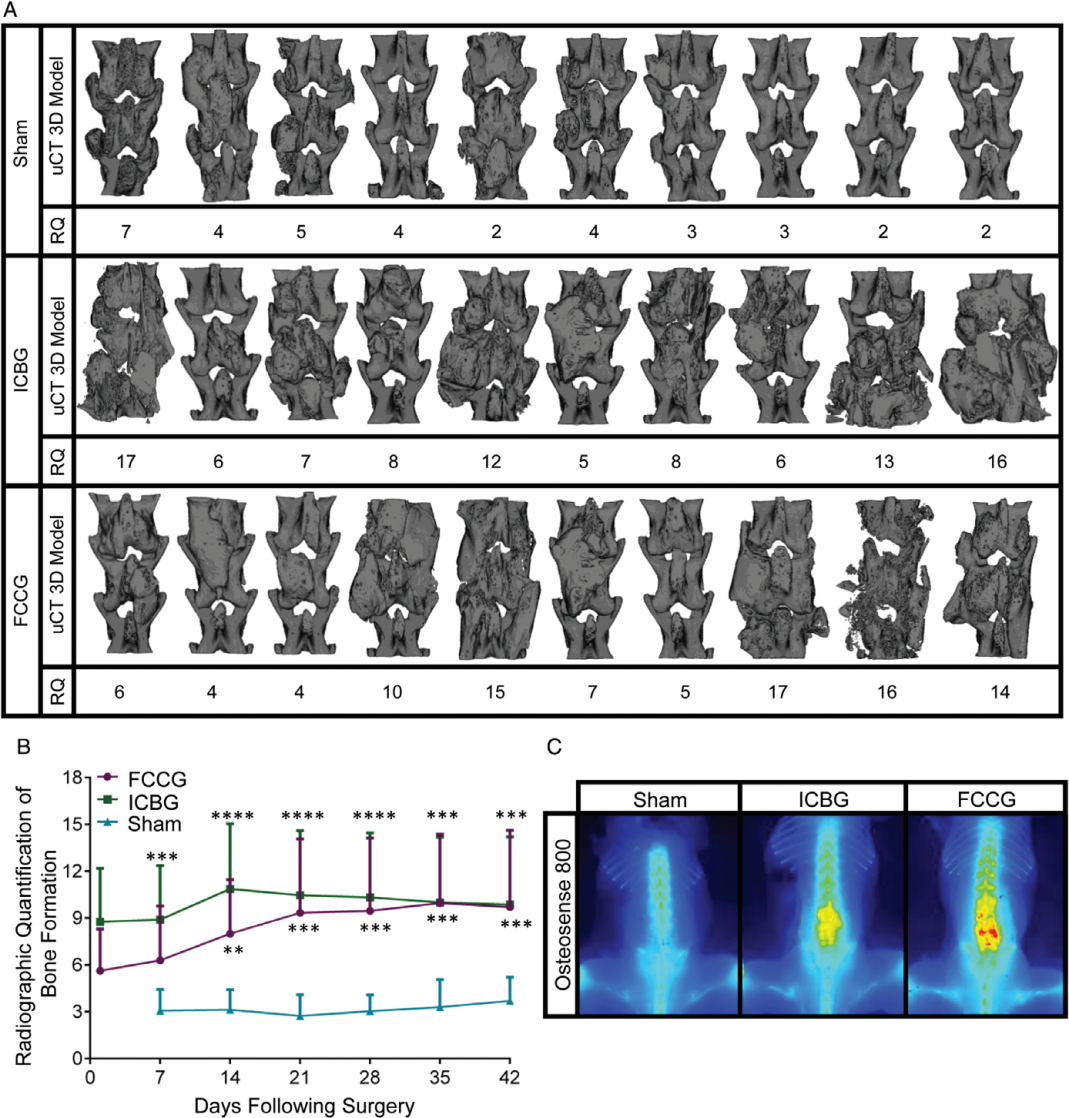
Implantation of hypertrophic chondrocytes promotes bone formation. (A) Three‐dimensional microcomputed tomography (3D μCT) reconstructions of the posterior spine (L3‐L5) following sham surgery, implantation of iliac crest bone graft (ICBG), or implantation of fracture callus chondrocyte graft (FCCG). Median radiograph quantification (RQ) per mouse correlates visually with the amount of bone formation (B) longitudinal RQ of bone formation via blinded scoring of digital radiographs (Figure S1). Points represent mean score between 3 reviewers per mouse ±SD. *N* = 10 mice per experimental group. *Statistical significance between ICBG or FCCG and sham. **P < 0.01, ***P < 0.001, ****P < 0.0001. Alpha = 0.05. No statistical difference between experimental groups was detected at any time point. (C) *in vivo* fluorescent imaging of bone deposition using Osteosense 800

3D renderings of L3 to L5 spinal levels by μCT revealed that both FCCG and ICBG increased bone formation at 6 weeks postsurgery to equivalent levels as compared to the sham control group (Figure 4A). Additionally, the median radiographic quantification scores per mouse (RQ) at 42 days postimplantation correlated visually with the amount of bone present (Figure 4A and Figure S1). As opposed to ICBG, in which bone was observed without changes in quantity over time, FCCG bone formation developed substantially over time and equaled that of ICBG by 21 days post‐implantation (Figure 4B). Furthermore, sensitive assessment of bone formation at 42 days post‐implantation demonstrated marked increases in newly formed bone, with the greatest amounts seen in mice receiving a FCCG.

While the primary objective of this study was to assess the ability of hypertrophic chondrocytes to promote bone formation in the posterolateral spine, we also observed by μCT analysis successful bony union of the laminae/transverse processes as well as a longitudinal bony bridge across vertebrae, indicative of fusion in a subset of animals from both the FCCG and ICBG group (Video S1). Taken together, these results support the hypothesis that FCCG can promote bone formation to comparable to ICBG, and therefore warrants further investigation as a potential graft alternative in spinal fusion.

## 4 DISCUSSION

To our knowledge, this is the first proof‐of‐concept study investigating the use of hypertrophic chondrocytes in promoting bone formation in the posterolateral spine. Ultimately, these findings support the hypothesis that hypertrophic chondrocytes have the capacity to drive posterolateral bone formation, with equal or greater efficacy as compared to ICBGs. Furthermore, while not a primary focus of this work, we observed successful bony bridging across vertebrae by μCT analysis, despite the lack of mechanical stabilization. While not designed to assess spinal fusion or fusion mass strength, these results are promising and warrant further investigation. Therefore, future studies aimed at (1) assessing spinal fusion following implantation of hypertrophic chondrocytes and (2) producing ample and clinically feasible sources of hypertrophic chondrocytes for testing in larger rodent and small animal studies are necessary to translate these findings to clinical use.

During fracture healing and spinal fusion, it is known that new bone formation takes place both by intramembranous and endochondral ossification.^13^, ^15^ Current strategies to promote vascularized bone regeneration have largely focused on the process of direct intramembranous bone formation while stimulating angiogenesis through the local application of growth factors.^10^, ^13^, ^22^, ^23^ VEGF,^24^ fibroblast growth factor (FGF‐2),^25^ platelet‐derived growth factor (PDGF),^19^ hydroxyapatite, BMPs,^23^, ^26^ and other factors are currently applied therapeutically. The problem with this direct intramembranous approach is that it is not very efficient at promoting neovascularization and/or successful graft incorporation, frequently resulting in early graft failure.^15^, ^27^, ^28^ Boden et al demonstrated that endochondral ossification occurs in the central/hypoxic zone of the fusion bed, which is also where pseudarthroses occur. Thus, strategies aimed at augmenting intramembranous bone formation may not be addressing the problem area in the fusion bed. Furthermore, Arthur Ham's work has demonstrated that osteoblasts cannot survive more than 200 μm from an oxygen source (vascular capillary).^29^ Thus, augmentation strategies that rely on the influx of osteoblasts into a relatively hypoxic fusion bed, like the direct intramembranous approach, are inherently limited.

In contrast, endochondral ossification forms new bone indirectly through a cartilaginous intermediary known to survive hypoxia, while simultaneously inducing angiogenesis. During long bone development, as well as fracture repair, neovascularized bone forms from a cartilage anlage under the direction of hypertrophic chondrocytes.^12^, ^16^, ^30^, ^31^ This process has the advantage of being highly angiogenic,^17^, ^28^, ^32^ while taking place through a progression of mesenchymal stem cell differentiation, vascularization, and mineralization. At the site of fracture healing, pluripotent stem cells differentiate into hypertrophic chondrocytes and recapitulate the developing physis in order to bridge the fracture gap with vascularized bone.^17^, ^30^ Importantly, hypertrophic chondrocytes have been shown to survive and proliferate in relative hypoxia (as compared to osteoblasts) while releasing required growth factors in proper temporal and spatial patterns.^17^, ^18^ Conveniently, these hypertrophic chondrocytes, for the sake of analogy, can be considered a conceptual “bone graft vesicle,” containing many of the necessary factors (ie, VEGF, BMP, and hydroxyapatite) to promote new bone formation in an area of relative hypoxia. To be sure, such factors are also present in iliac crest bone graft. In the present study, our findings suggest that hypertrophic chondrocytes may be used to induce bone formation in the spine.

Recently, rat studies have established the feasibility and efficacy of hypertrophic chondrocytes to induce new bone formation in tibial defects. Bahney et al showed that cartilage, isolated from a healing fracture callus, was effective at inducing vascularized bone formation in 2 mm defects of the tibia—confirmed by μCT and histology.^32^ The cartilage grafts in this previous study were shown to be equally as effective as the autograft, and superior to the allograft, in terms of both fusion rate and fusion strength. In this current study, a similar hypothesis was employed, and we found that hypertrophic chondrocytes augmented paraspinous bone formation comparable to standard ICBG. While the purpose of this study was not to assess the mechanism of bone formation (intramembranous vs endochondral ossification), use of this model in future studies in combination with lineage tracing experiments may prove insightful.

Although the findings of the current study provide a foundation for advancement in bone graft biologics, they are not without limitation. Primarily, the murine model utilized here is not directly applicable to human anatomy and/or physiology, and thus, directly extrapolating these results to human spinal fusion is not plausible. Rather, the findings here serve to establish the proof‐of‐concept foundation for potential transition to small and larger animal studies. Secondarily, use of a murine model precludes the use of pedicle screws or surgical stabilization to immobilized vertebral segments; yet even in an unstabilized setting, we did observe cortical bridging between vertebrae. Therefore, future studies in larger rodent and animal models, where pedicle screws/surgical stabilization can be employed, are warranted and should investigate the optimal mechanical stimulation of hypertrophic chondrocyte to promote maturation and ossification. Lastly, while this study establishes that hypertrophic chondrocytes may effectively augment bone formation in the posterolateral spine, soft‐tissue fracture calluses are not a feasible harvest source in clinical practice. Thus, this study provides the foundation for future work aimed at producing clinically practical sources of hypertrophic chondrocytes. One such promising avenue may be isolating pluripotent cells from circulation or periosteum and differentiating these cellular populations into hypertrophic chondrocytes *ex vivo*. Despite these limitations, we believe that the findings presented here provide proof‐of‐concept and establish a new paradigm in bone graft alternatives that will drive future research in larger animal models, and ultimately if successful, humans.

## 5 CONCLUSIONS

The findings of this proof‐of‐concept study, along with recent progress in tissue engineering, support the concept that hypertrophic chondrocytes can induce new bone formation around the spine with similar efficacy to that of ICBG. To our knowledge, the work presented here is the first to investigate the efficacy of hypertrophic chondrocytes in augmenting bone formation in the posterior region of the lumbar spine. These findings provide the foundation for future larger rodent and small animal studies to confirm our results and assess whether hypertrophic chondrocytes can induce spinal fusion at rates similar to ICBG with or without mechanical stability. If these results are recapitulated, investigations into the therapeutic potential of hypertrophic chondrocytes to augment bone formation clinically may be warranted.

## Supporting information

Figure S1 Radiological quantification of bone formation between transverse processes. (A) To assess the amount of newly formed bone between the transverse processes, the areas between L3, L4, and L5 vertebrae processes were delineated. First, the pedicle for each vertebra was identified. Second, a vertical line was drawn between the top of the proximal pedicle to the top of the adjacent pedicle to delineate the height of the medial boundary. Third, the height of the lateral boundary was delineated as the distance between the lateral edges of the transverse processes to the adjacent transverse processes. Finally, these 2 vertical lines were connected to form the analysis area. (B) and (C) Example images with subsequent scores for each delineated section.

Video S1 MicroCT evaluations of the posterolateral spine at 6 weeks following implantation. Views in the lateral to medial or dorsal to ventral orientation for mice that underwent sham surgery, ICBG, or FCCG implantation..
