# Supplementary figures and images for: Autologous chondrocyte grafting promotes bone formation in the posterolateral spine
Source: JOR Spine. 2018 Mar 23;1(1):e1001. doi: 10.1002/jsp2.1001 (PMC6686810; doi:10.1002/jsp2.1001)

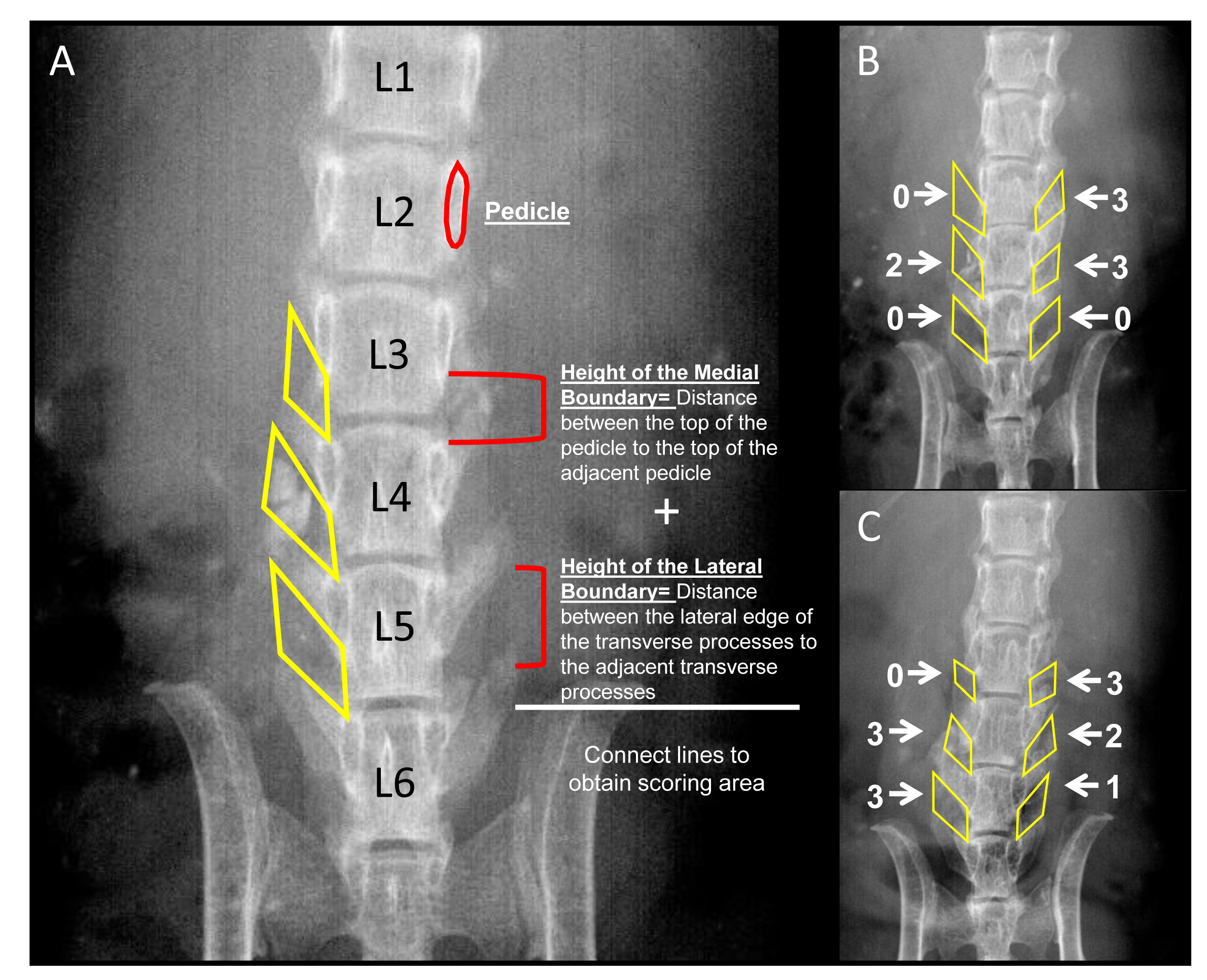

Supplement: Supplementary file 1 — Figure S1 Radiological quantification of bone formation between transverse processes. (A) To assess the amount of newly formed bone between the transverse processes, the areas between L3, L4, and L5 vertebrae processes were delineated. First, the pedicle for each vertebra was identified. Second, a vertical line was drawn between the top of the proximal pedicle to the top of the adjacent pedicle to delineate the height of the medial boundary. Third, the height of the lateral boundary was delineated as the distance between the lateral edges of the transverse processes to the adjacent transverse processes. Finally, these 2 vertical lines were connected to form the analysis area. (B) and (C) Example images with subsequent scores for each delineated section. [file JSP2-1-e1001-s001.tif]
